# Milk Oligosaccharides over Time of Lactation from Different Dog Breeds

**DOI:** 10.1371/journal.pone.0099824

**Published:** 2014-06-12

**Authors:** Shirin Macias Rostami, Thierry Bénet, Julie Spears, Arleigh Reynolds, Ebenezer Satyaraj, Norbert Sprenger, Sean Austin

**Affiliations:** 1 Nestle Research Center, Nestec S.A., Lausanne, Switzerland; 2 Nestle Purina Research, Nestec S.A., St. Louis, Missouri, United States of America; University of Sydney, Australia

## Abstract

The partnership of humans and dogs goes back to over 10'000 years, yet relatively little is known about a dog's first extra-uterine nutrition particularly when it comes to milk oligosaccharides. We set out to identify and quantify milk oligosaccharides over the course of lactation from different dog breeds (Labrador retriever, Schnauzer and 3 Alaskan husky crossbreeds). To this end, 2 different chromatographic methods with fluorescence and mass spectrometry detection were developed and one was validated for quantification. Besides lactose and lactose-sulphate, we identified 2 different trisaccharides composed of 3 hexose units, 3′sialyllactose (3′SL), 6′sialyllactose (6′SL), 2′fucosyllactose (2′FL), and a tetrasaccharide composed of 2 hexoses, an N-acetylhexosamine and a deoxyhexose. 3′SL was present at the highest levels in milk of all dog breeds starting at around 7.5 g/L and dropping to about 1.5 g/L in the first 10 days of lactation. 6′SL was about 10 times less abundant and 2′FL and the tetrasaccharide had rather varying levels in the milk of the different breeds with the tetrasaccharide only detectable in the Alaskan husky crossbreeds. The longitudinal and quantitative data of milk oligosaccharides from different dog breeds are an important basis to further our understanding on their specific biological roles and also on the specific nutritional requirements of lactating puppies.

## Introduction

In mammals, milk provides a source of key nutrients that act individually and jointly to insure optimal growth, development and protection of the neonate during its initiation and adaptation to the extra-uterine life. These nutrients include proteins, lipids, carbohydrates, minerals and vitamins. The precise composition of milk varies between different mammalian species and over the course of lactation representing a likely evolutionary adaptation to the respective species' life history and ecological niche.

Of the milk carbohydrates, the disaccharide lactose mostly represents the dominant form especially in eutherian mammals, but less so in monotremes and marsupials, where free oligosaccharides (OS) predominate [1]. Free oligosaccharides represent an interesting fraction of milk carbohydrates, varying by species and time of lactation, and exhibiting a diversity of structures and quantity. Generally, they are extensions of lactose by one or several monosaccharides such as galactose, *N*-acetylglucosamine, *N*-acetyl-galactosamine, sialic acid (*N*-acetyl-neuraminic acid, *N*-glycoly-neuraminic acid), and fucose.

In general, milk oligosaccharide structures are built on lactose, yet some were reported with a galactose at the reducing end instead of glucose [2]. Whether this is due to a secondary loss or a synthesis with galactose as first acceptor compound is not known. In the mammary glands oligosaccharide synthesis is carried out

by the action of specific glycosyltransferases and their site of expression and genetic polymorphism determine the oligosaccharide profiles found in milk of different species at specific times [3]–[6].

While lactose is digestible and a major energy source for the suckling newborn, non-digested milk OS were proposed to primarily modulate the establishing microbiota from oro-, nasopharyngeal cavity all along the gastrointestinal tract by acting as prebiotics for beneficial microbiota and as decoys for pathogens [7]–[9]. Despite this general notion, specific functions of individual oligosaccharide structures are not sufficiently understood and this is especially true when it comes to their contribution to the biology of specific mammalian species. Milk sialyloligosaccharides such as the sialyllactoses were proposed to act as a delivery vehicle of sialic acid for use in the synthesis of gangliosides and sialoglycoproteins especially in brain [10]–[12]. However, at least in mice, sialyllactoses were also shown to modulate the gut microbiota with their effects lasting beyond the suckling period [5]. Other oligosaccharide structures such as lactosamine-lactose (i.e. Lacto-N-tetraose, lacto-N-*neo*tetraose) were shown to act as prebiotics stimulating the growth of specific bacteria for example of the genus bifidobacteria [13]. Further, lacto-N-*neo*tetraose (LNnT) and fucosylated OS may inhibit or at least delay the adhesion of pathogenic bacteria to the mucosal epithelia. For example, 2′-fucosyllactose (2′FL) has been observed to reduce the load of *Campylobacter jejuni* [14].

The presence of OS in non-human mammals has been studied in the milk of a wide variety of both domestic and wild animals [1], [2], [15]. This is of particular interest when studying the evolutionary origins of milk and its constituents such as the free OS, but it is equally interesting when trying to understand the requirements of such constituents in relation to the ontogeny, life history and environmental niche of a particular species.

Several OS were previously identified from milk of different species from the order of the *Carnivora*.

From the suborder of the *Caniformia* these include bears [16]–[18], seals [19], [20] and *Canis lupus familiaris* (dog). In milk of a mongrel dog the formation of the OS fucosyllactose, 3′sialyllactose and 6′sialyllactose was reported [4] and in milk of a beagle dog 2′fucosyllactose and sialyllactose were identified together with sulphated lactose [21].

Here we report the partial characterization and quantification of milk OS from several dog breeds over the course of lactation. In order to do so, the oligosaccharides were labelled with 2-aminobenzamide then partially identified via LC-MS using two different gradients (co-eluting oligomers on one gradient could be separated on the other). Identities were confirmed by comparing retention times with those of known standards.

A quantitative method was then developed based on the gradient giving best separation of the major oligosaccharides, and it was validated prior to quantitative analysis of the oligosaccharides in the milk samples.

## Materials and Methods

### Materials

3′-Sialyllactose (3′SL) was obtained from Kyowa Hakko Kogyo Co, Ltd, Tokyo, Japan. 6′-Sialyllactose (6′SL) and 2′-fucosyllactose (2′FL) were obtained from Glycom A/S, Lyngby, Denmark. Lactose, sodium cyanoborohydride, 2-aminobenzamide, laminaritriose and dimethylsulphoxide were obtained from Sigma-Aldrich, Buchs, Switzerland. Water was deionised of 18.2 MΩ quality produced by a Milli-Q Plus system (Millipore, Billerica, MA). All other chemicals were obtained from Merck, Darmstadt, Germany.

### Milk Samples

The trial protocol for milk sampling was conducted in strict accordance with the guidelines established by the Nestle Purina Pet Care (NPPC) Advisory Committee and approved by the Nestle Purina animal care and use committee (reference number NT1550). Milk samples from 7 individual dogs were used in the present study: Three dogs were crosses of Alaskan Husky and German Shorthair Pointer; Annie, Uli and Isis (AH-GP). One was an Alaskan Husky – English pointer cross; Kirsten (AH-EP). One was an Alaskan Husky; Ella (AH). One was a Labrador Retriever (LR). One was a Miniature Schnauzer (SCH).

Milk was collected at eight different time points throughout the lactation period from day 0 (birth) to day 40 (on days 0, 1, 3, 5, 10, 20, 30, 40). To prepare for milk collection, lactating bitches were separated from their suckling pups for 2 hours before milking. Before milk collection, teats were cleaned by wiping with medicinal alcohol. Cleaned teat areas were air dried and milk was collected by gentle hand stripping of the teats. Milk from different teats of the same bitch was combined. Samples were immediately stored at −80°C until required for analysis.

For oligosaccharide identification and characterization, samples from one dog (Annie) were selected from the initial, middle and end stage of lactation (day 1, day 10 and day 40). During validation of the quantitative method, milk collected from Annie on day 10 of lactation was used for precision measurements, and for recovery experiments a pooled milk sample was prepared combining 1.5 mL of milk from Annie on day 20 of lactation with 1.5 mL of milk from Uli on day 30 of lactation and 1.5 mL from Uli on day 40 of lactation. The pooled milk was mixed in a plastic tube and divided in aliquots of 150 µL, which were immediately frozen at −80°C.

### Determination of Lactose

Lactose was analysed using a general method employed in our lab for testing of food products. Dog milk (100 µL) was dissolved in 80 mL of water and agitated at 70°C for 25 min. The solution was then made up to 100 mL in a volumetric flask. Particles were removed by centrifugation at 10000×g for 5 min and a portion of the supernatant transferred to a vial suitable for the LC autosampler. An aliquot (25 µL) was analysed on a high performance anion exchange chromatograph equipped with a pulsed amperometric detector (HPAEC-PAD, ICS 3000, Thermo, Sunnyville, USA) using a Carbopac PA20 column (3×150 mm, 6.6 µm, Thermo) held at 30°C. Lactose was eluted at 0.5 ml/min using a sodium hydroxide/sodium acetate gradient as follows: 0 min, 6 mM NaOH; 1 min, 6 mM NaOH; 12 min, 15 mM NaOH; 21 min, 85.2 mM NaOH +60 mM sodium acetate; 21.1 min 150 mM NaOH +500 mM sodium acetate; 26 min, 150 mM NaOH +500 mM; 26.1 min, 300 mM NaOH; 31 min, 300 mM NaOH; 31.1 min 6 mM NaOH; 37 min 6 mM NaOH. Under these conditions lactose has a retention time of approximately 18 min. Sodium hydroxide (300 mM, 0.2 mL/min) was added post-column to ensure sufficient hydroxide concentration for detection. Detection was performed by pulsed amperometry using a gold working electrode and a standard quadruple waveform for carbohydrates (t = 0 s, V = +0.1 V; t = 0.2 s, V = +0.1 V; t = 0.4 s, V = +0.1 V; t = 0.41 s, V = −2.0 V; t = 0.42 s, V = −2.0 V; t = 0.43 s, V = +0.6 V; t = 0.44 s, V = −0.1 V; t = 0.50 s, V = −0.1 V). Quantification was performed using an external calibration curve.

### Oligosaccharide Labelling

Samples were labelled with 2-aminobenzamide (2AB) using an adaptation of the method of Bigge et.al. [22] described by Bénet and Austin [23]. Briefly, an aliquot of milk (50 µL) was transferred to a microtube and laminaritriose solution (0.3 nmol/mL; 50 µL) was added. After mixing, an aliquot (20 µL) was transferred to a 2 mL microtube together with the 2AB labelling reagent (0.35 mol/L 2-AB and 1.0 mol/L sodium cyanoborohydride in dimethyl sulfoxide containing 30% acetic acid; 200 µL). The samples were incubated at 65°C for 2 h after which they were cooled on ice. A solution of acetonitrile/water (3/1 v/v; 1.5 mL) was added to the cooled solution before transferring to a vial suitable for the chromatography system. Solutions of standard OS were treated in the same way as the milk.

### Separation of 2-AB Labelled Oligosaccharides

Labelled OS were analysed using a Dionex Ultimate 3000RS Ultra High Performance Liquid Chromatography (UHPLC) system coupled to a Dionex RF2000 fluorescence detector (both Thermo, Sunnyville, USA) and a Q-Trap 4000 Mass spectrometer (AB Sciex, Framingham, MA). Separation was achieved on a BEH Glycan analytical column (2.1×150 mm, 1.7 µm, Waters, Milford, MA) using one of three different methods described below. For two of the methods (LC methods 1 and 2) a Van Guard BEH Amide guard column (1.7 µm; 2.1×5 mm, Waters) was used as a trapping column to capture the 2AB labelled OS and remove excess labelling reagents [23]. For the third method (LC method 3) excess reagents were not removed and a guard column was not employed. For methods 1 and 2 the system was controlled by Analyst version 1.5 (AB Sciex) with DCMS-link (Thermo) to control the chromatography system. Method 3 was used without the mass spectrometer and in this case the system was controlled by Chromeleon version 6.8 (Thermo).

### LC Method 1

Method 1 was a binary gradient using acetonitrile (100%) as eluent A and an aqueous solution of ammonium formate (50 mM, pH 4.4) as eluent B. The analytical column was kept at 18°C and the trapping column was at ambient temperature (typically 21–22°C). Flow rate was 0.3 mL/min and the fluorimeter used an excitation wavelength of 330 nm and an emission wavelength of 420 nm, gain was set at 1 and sensitivity to medium. The mass spectrometer was equipped with a turbo ion spray source operating in the negative ion mode. Parameters were set as follows: CUR: 12, GS1: 40, GS2: 20, IS −3750 V, TEM: 420°C, DP: −119, EP: −10.

Gradient conditions for method 1 were as follows. The sample solution (2 µL) was injected on the trapping column and washed for 5.75 minutes with 2% eluent B. Thereafter, flow was re-directed through both the trapping and the analytical columns and the eluent strength was increased with eluent B to 16% at 7 min, then 16% at 12.6 min, 39% at 36.4 min, 80% at 37 min, 80% at 40 min, followed by equilibration with 2% B from 41.5 min to 44.5 min. Then the valve was switched to direct flow through the trapping column only before the next injection (see Table S1 for more details).

### LC Method 2

Method 2 was a binary gradient using acetonitrile (100%) as eluent A and an aqueous solution of ammonium acetate (120 mM, pH 5.5) as eluent B. The analytical column was kept at 65°C and the trapping column was at ambient temperature (typically 21–22°C). The fluorimeter used an excitation wavelength of 330 nm and an emission wavelength of 420 nm, gain was set at 1 and sensitivity to medium. The mass spectrometer was equipped with a turbo ion spray source operating in the negative ion mode. Parameters were set as follows: CUR: 12, GS1: 62, GS2: 28, IS −3750 V, TEM: 420°C, DP: −110, EP: −10.

Gradient conditions for method 2 were as follows. The sample solution (2 µL) was injected on the trapping column and washed for 2.5 minutes with 5% B at 0.6 mL/min. Thereafter flow was re-directed through both the trapping and the analytical columns and the eluent strength was increased to 12% B at 3 min, then 12% B at 10 min, 16% B at 20 min, 16% B at 35 min, 34% B at 50 min, 80% B, 0.5 mL/min at 51 min, 80% B, 0.5 mL/min at 54 min, 10% B, 0.5 mL/min at 55 min, 10% B, 0.6 mL/min at 61 min, 5% B, 0.6 mL/min at 61.1 min, then the valve was switched to direct flow through the trapping column only before the next injection (see Table S2 for more details).

### LC Method 3

Method 3 was a shortened version of method 2 and it was used for quantitative analysis only. It was a binary gradient using acetonitrile (100%) as eluent A and an aqueous solution of ammonium acetate (120 mM, pH 5.5) as eluent B. The analytical column was kept at 65°C and the trapping column was not used. The fluorimeter used an excitation wavelength of 330 nm and an emission wavelength of 420 nm, gain was set at 1 and sensitivity to medium.

Gradient conditions for method 3 were as follows. The sample solution (1 µL) was injected on the analytical column and eluted with 12% B at 0.6 mL/min, then 12% B at 10 min, 16% B at 20 min, 16% B at 35 min, 80% B at 36 min, 80% B at 39 min, 12% B at 40 min, 12% B at 45 min, then the system was ready for the next injection (see Table S3 for more details).

### Validation of Method 3 for Quantification

Method 3 was validated in a single lab by testing linearity, recovery and precision (repeatability (r) and intermediate reproducibility (iR)). All data were analysed using robust statistics and an internal software package QStat.net.

### Linearity

Linearity was tested at two different concentration ranges for the oligosaccharide standards 2′FL, 3′SL, and 6′SL, from 500 to 6000 µM (high concentrations) and from 10 to 600 µM (low concentrations). All solutions were prepared in triplicate from 50 mM stock solutions of each oligosaccharide. Seven points were used to build the calibration curve in the high concentration range and nine points were used for the calibration curve in the low concentration range.

Initially, stock solutions (50 mM) were prepared for each individual oligosaccharide these were then mixed and diluted to produce the final solutions for linearity tests (Table S4 details the range and exact concentrations of the solutions). High and low concentration solutions were prepared in triplicate separately on different days and labelled according to the method described above. The analysis was performed using analytical method 3. Data was statistically analysed using the in-house software package Q-Stat.

### Trueness/Recovery

Trueness was determined using spike and recovery experiments. Three spike levels were considered: blank (with no addition of OS), low (with a spike of about 300 µmol/L of each oligosaccharide) and high (with a spike level of about 2000 µmol/L).

For low spike experiments, 50 µL of the 6000 µM solution used for linearity experiments were introduced in a 1 mL volumetric flask and then filled with the pooled milk up to 1 mL volume. In the high spike experiments, 40 µL of each individual oligosaccharide stock solution (50 mM) used for linearity experiments were introduced in a 1 mL volumetric flask and the flask was filled to the mark with pooled milk. The non-spiked pooled milk was also used as a “blank”.

Six samples from each solution (blank, low and high spike) were labelled according to the method described and analysed with UHPLC using method 3 described above. Calibration was performed using a seven point standard curve with oligosaccharide concentrations at approximately 200, 400, 600, 1000, 2000, 3000 and 4000 µM. The exact concentrations for each oligosaccharide are given in Table S4.

The data were analysed by QStat.net. The recovery for each oligosaccharide was determined by dividing the difference between the ‘oligosaccharide amount measured in spiked sample’ and ‘oligosaccharide amount measured in blank sample’ with the ‘theoretical amount of oligosaccharide spiked in to the blank’. A t-test with a 95% confidence interval was then used to assess if the measured recovery was significantly different from 100%

### Precision

To estimate method precision a sample of dog milk (from Annie at lactation day 10) was analysed in duplicate on 9 different days. Each day the milk was prepared in duplicate following the labelling protocol described, and analysed on the UHPLC using method 3. The duplicates data from each day were used to estimate method repeatability, and the data from the different days were used to estimate method intermediate reproducibility. All statistical analyses were carried out using the in-house software package QStat.net using robust statistics. The equations used by the software have been described previously [24].

### Quantification of Oligosaccharides

Milk OS from 5 different dogs were quantified at different stages of lactation from colostrum to the weaning stage of lactation. The milk donors were the dogs Ella, Isis, Kirsten, LR (Labrador retriever) and SCH (Schnauzer). Milk samples were stored frozen at −80°C in aliquots of 0.5 mL until analysed. All samples were labelled in duplicate according to the procedure described and analysed by UHPLC method 3. Quantification was performed using a 7 point standard curve containing approximately the following concentrations of each oligosaccharide: 200, 400, 600, 1000, 2000, 3000 and 4000 µM. The exact concentrations for each oligosaccharide are given in Table S1.

## Results

### Characterization of Milk Oligosaccharides

To characterize milk OS, we prepared a pooled milk sample with milk collected from different points during the lactation period collected from an Alaskan husky-German shorthair pointer dog named Annie. Using 2 different chromatographic methods coupled with mass spectrometry detection we found mass to charge ratios depicted in table 1 that correspond to (i) the disaccharides Hex2 (m/z 461.3), Hex-HexNAc (m/z 502.3) and Hex2-PO3H or Hex2-SO3 (m/z 541.3) and (ii) the OS Hex3 (m/z 623.4), dHex-Hex2 (m/z 607.4), Hex2-HexNAc-dHex (m/z 810.4), and NeuAc-Hex2 (m/z 752.4) (Table 1).

**Table 1:** Identification of oligosaccharides in dog milk samples over time of lactation using 2 different chromatographic methods coupled with mass spectrometry and comparison of retention times with genuine pure standards.

m/z	t_R(1)_ (min)	t_R(2)_ (min)	Composition	Identity	Day 1	Day 10	Day 40
461.3	18.2	9.0	Hex_2_	Lactose	•	•	•
541.3	17.5	9.2	Hex_2_ (SO_3_/PO_3_H)	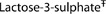	•	•	•
502.3	17.8	8.3	Hex HexNAc	nd	nd	•	•
607.4	21.2	11.5	Hex_2_ dHex	2′-Fucosyllactose*	•	•	•
623.4	24.4	19.7¥	Hex_3_	nd	•	•	•
752.4	25.1	23.4	Hex_2_ NeuAc	3′-sialyllactose*	•	•	•
810.4	25.1	20.9	Hex_2_ HexNAc dHex	Tetrasaccharide	•	•	•
623.4	25.8	nd¥	Hex_3_	nd	nd	•	•
752.4	27.4	29.9	Hex_2_ NeuAc	6′-sialyllactose*	•	•	•

m/z  =  mass to charge ratio of 2AB labelled oligosaccharide, tR(1)  =  retention time using method 1, tR(2)  =  retention time using method 2, Hex  =  hexose, HexNAc  =  *N*-acetylhexosamine, dHex  =  deoxyhexose, NeuAc  =  *N*-acetylneuraminic acid, NeuGc  =  *N*-glycolylneuraminic acid.

* Identification based on mass data and comparison of tR with genuine standard, 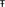 identification based on the fact that lactose-3-sulphate has previously been reported in dog milk by Bubb et.al. [21],

¥ two Hex_3_ oligosaccharides were separated by method one but only one peak is present when using method 2, so it is likely that they co-elute using method 2. nd  =  not detected/not determined.

• =  present.

**Figure 1:**
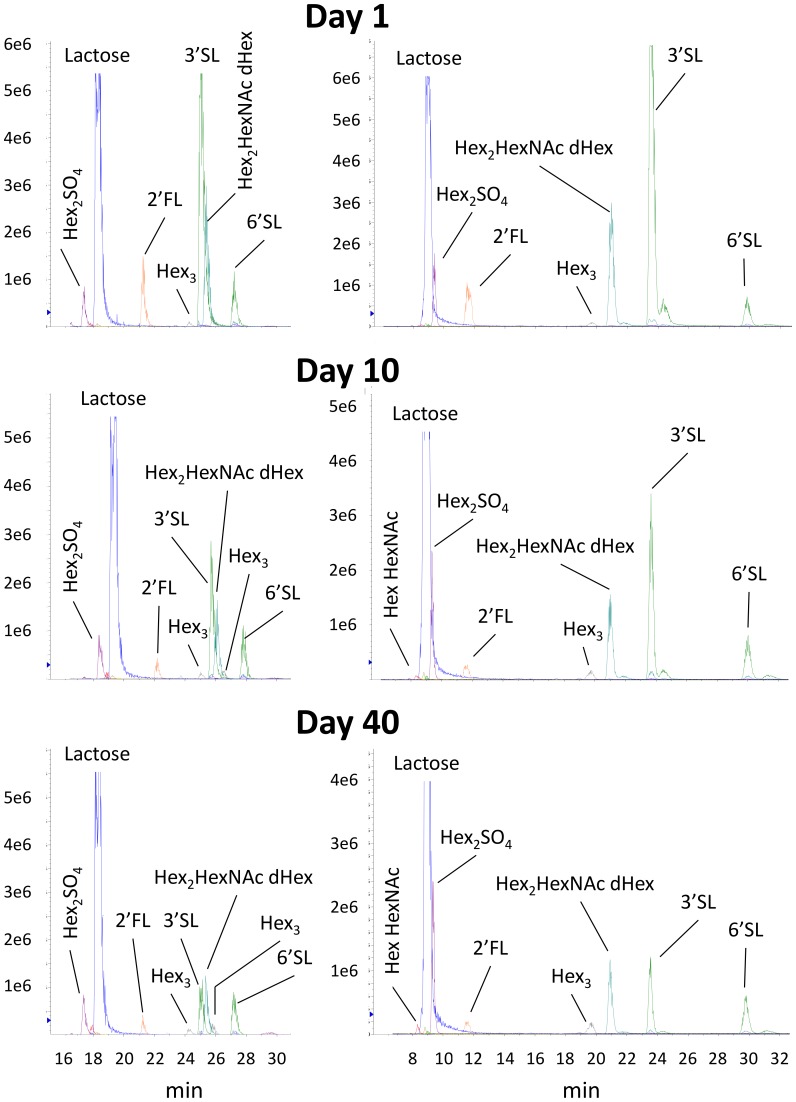
HPLC traces of milk oligosaccharides based on the *m/z* ratio signals at lactation day 1, 10 and 40 with HPLC method 1 (left hand side) and method 2 (right hand side).

We compared retention times of the disaccharides and OS seen in milk samples with those of authentic standard compounds. Using 2 different chromatographic methods signals in the milk sample were found to have identical retention times as lactose, 2′fucosyllactose (2′FL; dHex-Hex2 (m/z 607.4)), 3′sialyllactose (3′SL; NeuAc-Hex2 (m/z 752.4)) and 6′sialyllactose (6′SL; NeuAc-Hex2 (m/z 752.4)) (Figure 1; Table 1). Lactose-3-sulfate has been previously reported in dog milk [21] and therefore we assume the compound with the m/z of 541.3 to represent lactose-3-sulfate (Hex2SO3). In milk from lactation day 10 and 40 we found compounds corresponding to Hex3 at 2 different retention times when using the chromatography method 1. This indicates that at least 2 different Hex3 structures are present in dog milk (Figure 1; Table 1).

### Validation

Prior to quantification of the OS in dog milk, the method was validated for the OS which were available in our lab as quantitative standards (i.e. 2′FL, 3′SL and 6′SL). During the validation it was confirmed that each oligosaccharide had a linear response in the detector, that the method was reproducible in our laboratory (repeatability and intermediate reproducibility) and that the measurements were accurate.

*Linearity* was studied over a concentration range from 10 to 6000 µM. To handle such a wide range, data were split in to two overlapping ranges, a low range from 10–600 µM and a high range from 500–6000 µM. The highest point (6000 µM) was out of the linear range for all OS, so the maximum concentration in the linear range was defined as 5000 µM. R-squared values for the curves were between 0.98 and 0.99 and the plots of residuals were randomly distributed supporting the hypothesis that the response of the fluorescence detector used here was linear in the ranges under investigation.

#### Limit of detection (LoD) and quantification (LoQ)

The baseline noise was measured in the chromatograms of standards and also in some samples. The noise level of the baseline was consistently between 0.3–0.4 mV. LoD and LoQ were calculated using the data acquired for linearity to estimate the concentration of analyte required to result in a peak having a height equivalent to three times the baseline noise (1.2 mV) for LoD and 10 times the baseline noise (4.0 mV) for LoQ. For 2′FL the LoD and LoQ were 32 µM and 64 µM respectively. For 3′SL the LoD and LoQ were 41 µM and 88 µM respectively. For 6′SL the LoD and LoQ were 71 µM and 140 µM respectively.

*Trueness* was assessed by spike-recovery experiments. The recoveries obtained from spiking experiments at high level (around 2000 µM) were between 99–104%, and with the exception of the result for 2′FL were not significantly different from 100% when analysed using robust statistics (Table 2). At the lower spiking level (around 200 µM) recoveries were between 82–91% and were considered significantly different from 100% using robust statistics. The FDA recommendations for bioanalytical methods (U.S. Department of Health and Human Services, Food and Drug Administration (2001), Guidance for Industry: Bioanalytical Method Validation. Available: http://www.fda.gov/downloads/Drugs/GuidanceComplianceRegulatoryInformation/Guidances/UCM070107.pdf. Accessed 12 June 2013) propose that such methods should have recoveries in the region of 85–115%. All our analytes at both concentration levels were within the recommended recovery range, except 3′SL at the low level (200 µM) which has an 82% recovery. In the case of 3′SL the native concentration of the spiked sample is already quite high (650 µM) and the poor recovery may be linked to the fact that the additional spike level is quite low compared to the native content. In fact the levels of 3′SL in the milks analysed in this study were never below 50 µM.

**Table 2:** Dog milk trueness results.

Sample	Units	number of replicates	Median of results	Recovery %	SD (Rec)	Rec = 100%: Y/N
2′FL	µM	6	2070	104	0.010	N
3′SL	µM	6	1986	99.2	0.012	Y
6′SL	µM	6	2064	103	0.012	Y
2′FL	µM	6	271.4	90.5	0.018	N
3′SL	µM	6	247.3	82.2	0.039	N
6′SL	µM	6	271.5	90.2	0.049	N

*Precision* was assessed by analysing a dog milk sample in duplicate on nine different days. The data obtained were used to assess the method repeatability (RSD(r)) and intermediate reproducibility (RSD (iR)) (Table 3). Relative standard deviation of repeatability (RSD(r)) assessed using robust statistics was between 5.2–8.1% which is below the 15% level recommended by the FDA for the performance of bioanalytical methods. The RSD (iR) calculated using robust statistics ranged from 7.7–11% which are also below the 15% limit recommended by the FDA for such methods.

**Table 3:** Robust RSD_(r)_ and RSD_(iR)_ of 2′FL, 3′SL and 6′SL in dog milk from data measured in duplicates during 9 days.

OS	RSD_(r)_ %	RSD_(iR)_%
2′FL	5.2	10.5
3′SL	7.3	8.4
6′SL	8.1	7.7

### Quantification of Milk Oligosaccharides

**Figure 2:**
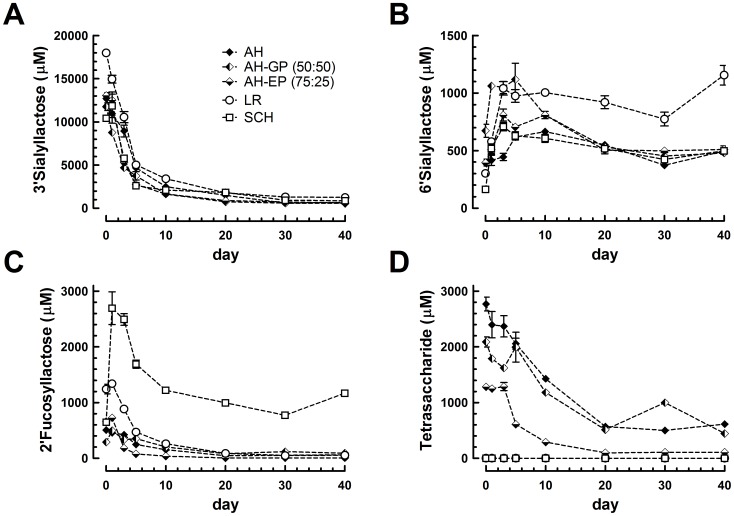
Levels of major oligosaccharides over time of lactation in milk samples from 5 different dog breeds (Alaskan husky (AH); Alaska husky German pointer (AH-GP); Alaskan husky English pointer (AH-EP); Labrador retriever (LR); Schnauzer (SCH)). A. 3′Sialyllactose; B. 6′Sialyllactose; C. 2′Fucosyllactose; D. Tetrasaccharide A (blood group A antigen type 5). Mean values with standard deviation of 2 replicate analysis are shown per milk and timepoint.

The OS 3′SL, 6′SL and 2′FL were quantified in milk samples of 3 Alaskan huskies (AH, AH-GP, AH-EP), 1 Labrador retriever (LR) and 1 Schnauzer (SCH) using external standard curves of authentic standard compounds. Since we did not have a quantitative standard, the tetrasaccharide (Hex2-HexNAc-dHex) was quantified using the 2′FL standard curve (Figure 2) assuming equimolar responses for the labeled OS. 3′SL was found to be by far the most prominent dog milk oligosaccharide. In the first days of lactation it was present at levels around 7.5 g/L (12 mM), which dropped rapidly to approximately 1.5 g/L at 10 days of lactation. During the further course of lactation, levels dropped further to around 0.6 g/L (Figure 2A). On the other hand, 6′SL levels reached about 0.3 to 0.6 g/L (0.5-1 mM) within the first days of lactation and reached a peak level at 5 days of lactation or remained at a rather constant level throughout lactation (Figure 2B). Of the dog breeds analyzed here, Schnauzer milk had highest 2′FL levels showing a maximum of approximately 1.2 g/L (ca. 2.5 mM) at around 2–4 days of lactation, thereafter levels dropped to approximately 0.5 g/L (Figure 2C). The other dog breeds had lower levels, yet they also had a peak 2FL level (0.3–0.5 g/L) during the first 5 lactation days followed by a drop within the first 10 days to around 0.1 g/L. The tetrasaccharide (Hex2-HexNAc-dHex) was only found in the Alaskan husky breeds and not in the Labrador retriever and Schnauzer. In early milk, up to 5 days of lactation, we estimate about 1.4 g/L (ca. 2 mM) of tetrasaccharide were present and by 20 days the levels fell below 0.5 g/L (Figure 2D).

### Quantification of Lactose

**Figure 3:**
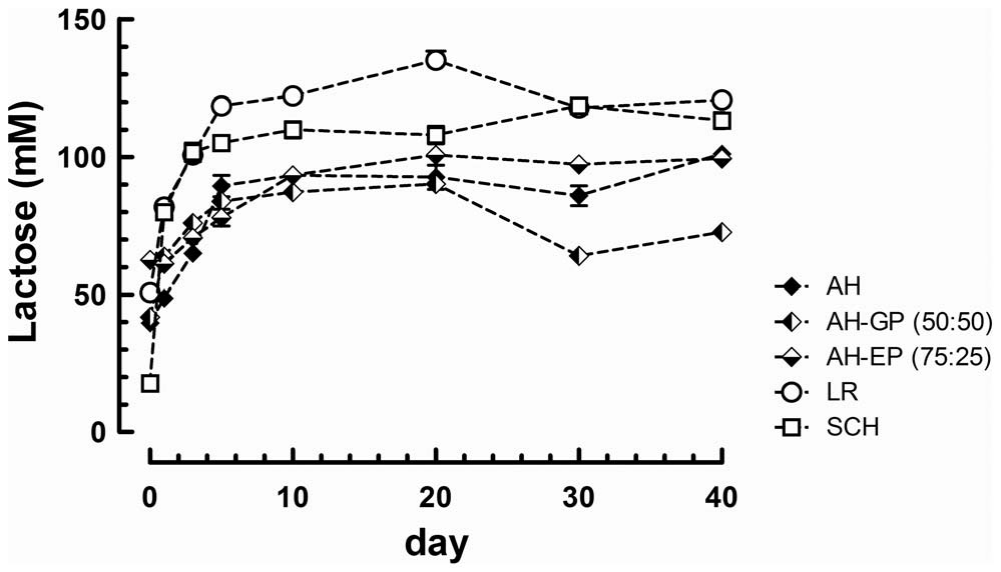
Lactose levels over time of lactation in milk samples from 5 different dog breeds (Alaskan husky (AH); Alaska husky German pointer (AH-GP); Alaskan husky English pointer (AH-EP); Labrador retriever (LR); Schnauzer (SCH)). Mean values with standard deviation of 2 replicate analysis are shown per milk and timepoint.

Lactose was quantified over the course of lactation in milk samples of 3 Alaskan huskies (AH, AH-GP, AH-EP), 1 Labrador retriever (LR) and 1 Schnauzer (SCH) using an external standard curve of lactose. In all milks analyzed lactose concentration increased during the first 5 days of lactation reaching up to around 80 to 120 mM, which corresponds to around 30 to 40 g/l (Figure 3). Thereafter, lactose levels in milk remained relatively constant over the rest of the lactation period that we monitored. Of note, the Alaskan huskies showed about 20% lower lactose levels compared to the Labrador retriever and Schnauzer.

## Discussion

Following the analysis of milk from different dog breeds over the course of lactation we confirm the presence of the milk OS 3′- and 6′sialyllactose (3′SL, 6′SL) and 2′fucosyllactose (2′FL) [4], [21]. We also have evidence to support the presence of lactose-3-sulphate in canine milk. To our knowledge for the first time, we further identified the presence of OS having masses corresponding to a disaccharide of the composition Hex HexNAc, two trisaccharides of the composition Hex_3_, and a tetrasaccharide of the composition Hex_2_ HexNAc dHex in canine milk. We also believe this to be the first quantitative study of the 3 major dog milk trisaccharides 3′SL, 6′SL, 2′FL and semi-quantitation of the tetrasaccharide Hex2 HexNAc dHex over the whole lactation period.

2′FL and Isoglobotriose (a Hex3 oligosaccharide: Gal(α1,3)Gal(β1,4)Glc) appear to be common components of the milk of members of the Caniformia suborder [15]. Of the milks analysed to date, the milk of the giant panda seems to be the only one lacking 2′FL, while isoglobotriose is absent only in the milk of the seals. Given the apparent conservation of isoglobotriose in the milks of this suborder it seems likely that one of the unidentified Hex3 OS in dog milk could be isoglobotriose, although many other oligosaccharides with the composition Hex3 are possible, including 3′-, 4′- and 6′-galactosyllactose. Unusually, since they seem to be quite abundant in many types of milk, the sialyllactoses are not widely reported in the milks of the *Caniformia* suborder. Only the milk of the giant panda is reported to contain both 3′- and 6′sialyllactose, and 3′sialyllactose has also been detected in the milk of the Japanese black bear [15] and polar bear colostrum, but not mature milk [25]. The milks of the other members of the suborder, namely seals, have either no acidic oligosaccharide reported, or they are larger OS (with the exception of dog milk). Of course it is possible that the studies performed on those milks have focused on the neutral oligosaccharide fractions, and thus the acidic OS have simply been underreported. With the current, yet limited, data on *Caniformia* milk, one might however speculate that 3′SL levels in milk could be associated with the relative altricial growth and development of dogs and bears compared to the more precocial seals.

In the milk of the different dog breeds analyzed here, 3′SL levels were the highest measured reaching levels of 10 to 18 mM or 6.3 to 11 g/L in milk of the first days. At 10 days of lactation 3′SL concentration dropped to about 1.5 g/L. Concomitant with the drop in 3′SL we observed lactose in milk to increase from 20–50 mM to 80–120 mM. In beagle milk, lactose concentrations were previously reported to increase from 16 g/L (46 mM) to 34 g/L (100 mM) in the first week of lactation [26], similar to the lactose levels we report here. Both with respect to volume and to lactose concentration the level of 3′SL in dog milk is relatively high, similar to the amounts described in mouse and rat milk [5], [27].Especially during the first week postpartum the high 3′SL concentration and 3′SL to lactose ratio might indicate a specific physiologic need of the newborn puppies. 3′SL seems to be an evolutionary conserved milk oligosaccharide, as it is present in most milk described to date thus suggesting that it has a specific biological function common to most mammals even if it seems to be a rare occurrence in the suborder *Caniformia*. With observation data in hand as reported here, further studies are needed to pinpoint the underlying biology. From mouse studies with sialyltransferase null mutant mouse dams showing reduced milk 3′SL levels and fostering wildtype pups [5], [28], we might speculate that also in dog puppies 3′SL aids in orchestration of the gut environment by modulation of specific gut microbiota establishment and mucosal immunity. Beyond, 3′SL might also serve as sialic acid carrier providing fuel for glycoslytion processes, possibly in particular *via* a boost of the N-acetyl-glucosamine route [12] and by fueling brain sialylation and function [10]. Especially, the latter might happen *in utero* for more precocial species, while more altricial animals might rely on specific milk components such as 3′SL to this end.

A high milk OS to lactose ratio was previously reviewed from an evolutionary perspective for other species of the Canoidea and related to the appearance and rate of lactose synthesis [1]. In dog milk we observed a high OS to lactose ratio especially in the first 5 days of lactation while lactose concentration increases and major OS concentration decreases. From a mechanistic viewpoint this might be linked to changes in lactose synthesis rates or to reduced expression or activity of glycosyltransferases in the mammary glands. From a physiologic viewpoint the high OS to lactose ratio in the very first days of lactation might be linked to the dog′s life history and reproductive physiology. While some milk components, such as lactose, primarily provide energy for growth and development, the non-digestible milk OS provide clues and fuel for the establishing gut microbiota and protection from infections [7]–[9]. Since dogs are born relatively immature their need might be elevated during the first 5 days of lactation for specific OS serving as conditional nutrients, and protective and microbiota orchestrating agents.

Lactose-3-sulphate was not quantified in this study since the method selected for quantitative analysis did not resolve the peak from that of lactose. However the LC-MS data collected from the milk of Annie (Fig 1) suggests that it could be the next most abundant oligosaccharide after 3′SL at day 10 of lactation and may be the most abundant non-lactose oligosaccharide in the later stages of lactation.

Generally, early dog milk showed highest concentration of OS similar to observations done with other mammals such as rat [27], [29], mouse [5], cow [30] and humans [31], [32]. Yet differences regarding specific OS exist. For example in rat milk 3′SL and 6′SL increase over the first 5 days of lactation [27], while in dog milk 3′SL levels are high right from the beginning of lactation, while 6′SL increases over the first couple of days. It is likely that the varying amounts of OS in milk point to specific needs of the suckling newborn over the course of lactation. When comparing the gross composition of canine milk with milk from other species, there appears to be a striking similarity to rat milk [33]. In milk from dogs, fat is reported at 9.5%, protein at 7.5% and lactose at 3.4%. In comparison milk from rats contains fat at 8.8%, protein at 8.1% and lactose at 3.4%. Now this similarity also extends to the levels and profile of 3′SL and 6′SL between milk from dog and rats. The question remains, what are the specific reproductive and developmental needs that are addressed by the unique composition of canine milk. Does evolution and environment influence the variation in milk composition?

In canine milk we identified a somewhat exotic milk oligosaccharide with an m/z ratio corresponding to the tetrasaccharide Hex2-HexNAc-dHex. Most likely this canine milk oligosaccharide corresponds to the oligosaccharide GalNAc-α-1,3-(Fuc-α-1,2-)Gal-β-1,4-Glc, commonly known as blood group A antigen type 5, or tetrasaccharide A. Indeed a commercial standard of blood group A antigen type 5 (Elicityl, France) co-migrates with one of the major dog milk OS upon separation by high performance anion exchange chromatography on a CarboPac PA1 analytical column (Thermo), which strongly suggests that the identified Hex2-HexNAc-dHex corresponds to GalNAc-α-1,3-(Fuc-α-1,2-)Gal-β-1,4-Glc (Figure S1). To the best of our knowledge this is the only milk oligosaccharide that has been reported having such a composition but it is not commonly found. To date it has only been reported in the milks of polar bear [20], lion [34], leopard [34], minke whale [35] and skunk [36] and as a minor component in the milk of humans having blood group A [37].

Interestingly, the fucosylated OS (tetrasaccharide A and 2′FL) show much higher variability between the different dog breeds analysed here than the sialyllactoses (3′SL and 6′SL). While the Alaskan husky breeds showed lowest 2′FL levels, they were the only breed with the fucosylated tetrasaccharide A. This suggests that in the Alaskan husky breed the 2′FL is further decorated with an N-acetyl-hexosamine by a GalNAc transferase activity, leading to lower 2′FL levels and the appearance of the fucosylated tetrasaccharide. In cow milk, presence of GalNAc-α-1,3- Gal-β-1,4-Glc is reported [38] indicating the presence of a GalNAc transferase activity similar to the one expected in Alaskan husky dog breeds. Kobata (2010) [39] reported the presence of such α-GalNAc containing OS, including the tetrasaccharide GalNAc-α-1,3-(Fuc-α-1,2-)Gal-β-1,4-Glc in the milk of human secretors of blood type A and such α-GalNAc containing fucosyl-OS were reported also in suckling infant feces [40].

## Conclusions

We have presented evidence confirming the presence of 2′FL, 3′SL, 6′SL and (to a lesser degree) lactose-3-sulphate in canine milk. In addition, and for the first time, we report the presence of a disaccharide having the composition HexNAc Hex, two trisaccharides of the composition Hex_3_ and a tetrasaccharide of the composition Hex_2_ HexNAc dHex postulated to be tetrasaccharide-A (also known as blood group A antigen type 5). We have shown that milk composition depends to some extent on breed, since the tetrasaccharide was only observed in Alaskan huskys or crosses thereof and that concentrations of the individual OS vary during lactation (more or less following the same pattern between the different breeds although the actual concentrations may vary significantly). The presented structural and longitudinal information on milk OS will help to further the insight into functional milk properties and specific needs of suckling newborns.

## Supporting Information

Figure S1 HPAEC-PAD profile of Alaskan Husky dog milk oligosaccharides and authentic standard blood group A antigen type 5 (Tetrasaccharide A).
(DOCX)

Table S1 Elution conditions for LC method 1.
(DOCX)

Table S2 Elution conditions for LC method 2.
(DOCX)

Table S3 Elution conditions for LC method 3.
(DOCX)

Table S4 Concentrations of standard oligosaccharide solutions used in the study.
(DOCX)
