# Supplementary material for: Milk Oligosaccharides over Time of Lactation from Different Dog Breeds
Source: PLoS One. 2014 Jun 12;9(6):e99824. doi: 10.1371/journal.pone.0099824 (PMC4068735; doi:10.1371/journal.pone.0099824)
Supplement: Figure S1 — HPAEC-PAD profile of Alaskan Husky dog milk oligosaccharides and authentic standard blood group A antigen type 5 (Tetrasaccharide A). (DOCX) [file pone.0099824.s001.docx]

***Figure S1.*** *HPAEC-PAD profile of Alaskan Husky dog milk oligosaccharides and authentic standard blood group A antigen type 5 (Tetrasaccharide A).*
