# Supplementary material for: Milk Oligosaccharides over Time of Lactation from Different Dog Breeds
Source: PLoS One. 2014 Jun 12;9(6):e99824. doi: 10.1371/journal.pone.0099824 (PMC4068735; doi:10.1371/journal.pone.0099824)
Supplement: Table S1 — Elution conditions for LC method 1. (DOCX) [file pone.0099824.s002.docx]

Table S1: Elution conditions for LC method 1

| Time (min) | Flow (mL/min) | %A | %B | Valve Position | Comment |
| --- | --- | --- | --- | --- | --- |
| 0.0 | 0.3 | 98 | 2 | 6-1 | Inject Sample & start sample clean-up |
| 5.7 | 0.3 | 98 | 2 | 6-1 | End of sample clean-up |
| 5.75 | 0.3 | 98 | 2 | 1-2 | Divert flow on to analytical column |
| 7.0 | 0.3 | 84 | 16 | 1-2 | Start isocratic elution of OS |
| 12.6 | 0.3 | 84 | 16 | 1-2 | End isocratic elution– start gradient |
| 36.4 | 0.3 | 61 | 39 | 1-2 | End gradient |
| 37 | 0.3 | 20 | 80 | 1-2 | Start column wash |
| 40 | 0.3 | 20 | 80 | 1-2 | End column wash |
| 41.5 | 0.3 | 98 | 2 | 1-2 | Start column re-equilibration |
| 44.45 | 0.3 | 98 | 2 | 1-2 | End column re-equilibration |
| 44.5 | 0.3 | 98 | 2 | 6-1 | Switch valve ready for next sample |
| Eluent A = Acetonitrile (100%). Eluent B = Ammonium Formate (50mM) pH 4.4. For valve plumbing scheme see Bénet & Austin (2011) Anal.Biochem. 414:166-168 doi: [10.1016/j.ab.2011.03.002](http://dx.doi.org/10.1016/j.ab.2011.03.002) | | | | | |
