# Supplementary material for: Milk Oligosaccharides over Time of Lactation from Different Dog Breeds
Source: PLoS One. 2014 Jun 12;9(6):e99824. doi: 10.1371/journal.pone.0099824 (PMC4068735; doi:10.1371/journal.pone.0099824)
Supplement: Table S2 — Elution conditions for LC method 2. (DOCX) [file pone.0099824.s003.docx]

Table S2: Elution conditions for LC method 2

| Time (min) | Flow (mL/min) | %A | %B | Valve Position | Comment |
| --- | --- | --- | --- | --- | --- |
| 0.0 | 0.3 | 95 | 5 | 6-1 | Inject Sample & start sample clean-up |
| 2.45 | 0.3 | 95 | 5 | 6-1 | End of sample clean-up |
| 2.5 | 0.6 | 95 | 5 | 1-2 | Divert flow on to analytical column & increase flow |
| 3.0 | 0.6 | 88 | 12 | 1-2 | Start isocratic elution of OS |
| 10.0 | 0.6 | 88 | 12 | 1-2 | End first isocratic step start first gradient |
| 20.0 | 0.6 | 84 | 16 | 1-2 | End first gradient, start second isocratic step |
| 35.0 | 0.6 | 84 | 16 | 1-2 | End second isocratic step – start second gradient |
| 50.0 | 0.6 | 66 | 34 | 1-2 | End second gradient |
| 51.0 | 0.5 | 20 | 80 | 1-2 | Start column wash |
| 54.0 | 0.5 | 20 | 80 | 1-2 | End column wash |
| 55.0 | 0.5 | 90 | 10 | 1-2 | Start column re-equilibration |
| 61.0 | 0.6 | 90 | 10 | 1-2 | End column re-equilibration |
| 61.1 | 0.6 | 95 | 5 | 6-1 | Switch valve & increase %A ready for next injection |
| Eluent A = Acetonitrile (100%). Eluent B = Ammonium Acetate (120mM) pH 5.5. For valve plumbing scheme see Bénet & Austin (2011) Anal.Biochem. 414:166-168 doi: [10.1016/j.ab.2011.03.002](http://dx.doi.org/10.1016/j.ab.2011.03.002) | | | | | |
