# Supplementary material for: Milk Oligosaccharides over Time of Lactation from Different Dog Breeds
Source: PLoS One. 2014 Jun 12;9(6):e99824. doi: 10.1371/journal.pone.0099824 (PMC4068735; doi:10.1371/journal.pone.0099824)
Supplement: Table S3 — Elution conditions for LC method 3. (DOCX) [file pone.0099824.s004.docx]

Table S3: Elution conditions for LC method 3

| Time (min) | Flow (mL/min) | %A | %B | Comment |
| --- | --- | --- | --- | --- |
| 0.0 | 0.6 | 88 | 12 | Inject Sample start isocratic elution |
| 10.0 | 0.6 | 88 | 12 | End first isocratic step start first gradient |
| 20.0 | 0.6 | 84 | 16 | End first gradient, start second isocratic step |
| 35.0 | 0.6 | 84 | 16 | End second isocratic step |
| 36.0 | 0.6 | 20 | 80 | Start column wash |
| 39.0 | 0.6 | 20 | 80 | End column wash |
| 40.0 | 0.6 | 88 | 12 | Start column re-equilibration |
| 45.0 | 0.6 | 88 | 12 | End column re-equilibration |
| Eluent A = Acetonitrile (100%). Eluent B = Ammonium Acetate (120mM) pH 5.5. | | | | |
