# Supplementary material for: Milk Oligosaccharides over Time of Lactation from Different Dog Breeds
Source: PLoS One. 2014 Jun 12;9(6):e99824. doi: 10.1371/journal.pone.0099824 (PMC4068735; doi:10.1371/journal.pone.0099824)
Supplement: Table S4 — Concentrations of standard oligosaccharide solutions used in the study. (DOCX) [file pone.0099824.s005.docx]

Table S4: Concentrations of standard oligosaccharide solutions used in the study

| Solution | Target Conc. (nmol/mL) | Actual Conc. (nmol/mL) | | |
| --- | --- | --- | --- | --- |
|  |  | 2’FL | 3’SL | 6’SL |
| Stock | 50000 | 50000 | 50050 | 50142 |
| Standard mix | 6000 | 6000 | 6006 | 6017 |
| Standard mix | 5000 | 5000 | 5005 | 5014 |
| Standard mix | 4000 | 4000 | 4004 | 4011 |
| Standard mix | 3000 | 3000 | 3003 | 3009 |
| Standard mix | 2000 | 2000 | 2002 | 2006 |
| Standard mix | 1000 | 1000 | 1001 | 1003 |
| Standard mix | 600 | 600.6 | 601.3 | 602.8 |
| Standard mix | 500 | 500.5 | 501.1 | 502.3 |
| Standard mix | 400 | 400.4 | 400.9 | 401.9 |
| Standard mix | 300 | 300.3 | 300.7 | 301.4 |
| Standard mix | 200 | 200.2 | 200.5 | 200.9 |
| Standard mix | 100 | 100.1 | 100.2 | 100.5 |
| Standard mix | 50 | 50.05 | 50.11 | 50.23 |
| Standard mix | 20 | 20.02 | 20.04 | 20.09 |
| Standard mix | 10 | 10.01 | 10.02 | 10.05 |
